# Freestyle aortic root prosthesis in combination with aortic replacement and open anastomosis: a retrospective analysis

**DOI:** 10.1186/s13019-021-01562-3

**Published:** 2021-06-26

**Authors:** Alicja Zientara, Kim Rosselet-Droux, Hans Bruijnen, Dragan Odavic, Michele Genoni, Omer Dzemali

**Affiliations:** 1 Department of Cardiothoracic Surgery, Royal Brompton and Harefield Hospital, Sydney Street, London, SW3 6NP UK; 2 University of Zurich, Rämistrasse 71, 8001 Zurich, Switzerland; 3 Department of Vascular Surgery, City hospital Augsburg, Stenglinstraße 2, 86156 Augsburg, Germany; 4 Department of Cardiac Surgery, Triemli City hospital Zurich, Birmensdorferstrasse 497, 8063 Zurich, Switzerland; 5 Rehabilitation Clinic Seewis, Cardiac Rehabilitation, Schlossstrasse 1, 7212 Seewis, Switzerland

**Keywords:** Freestyle prosthesis, Root replacement, Hemiarch replacement, Open arch anastomosis, Axillary cannulation, Selective brain perfusion

## Abstract

**Background:**

The Freestyle® bioprosthesis is used for pathologies of the aortic root. Additional resection of the ascending aorta and the proximal arch in dissections or aneurysms might be indicated. The aim was to assess mid-term outcome regarding prosthetic performance, stroke, reoperations, and survival in various pathologies comparing patients with and without additional procedures on the ascending aorta and proximal arch focusing on the standardised technique of unilateral antegrade cerebral perfusion under moderate hypothermia.

**Methods:**

Retrospective data analysis of 278 consecutive patients after Freestyle® root replacement between September 2007 and March 2017. Patients were divided in three categories due to the pathology of the aortic root (re-operation vs endocarditis vs dissection). Two groups based on the aortic anastomosis technique (open arch anastomosis (OA) versus non-open arch anastomosis (non-OA) were compared (119 OA vs 159 non-OA). Cardiovascular risk, previous cardiac events, intra- and postoperative data were evaluated. Inferential statistics were performed with Mann-Whitney U-test. Nominal and categorical variables were tested with Fisher-Freeman-Halton exact test. Kaplan-Meier estimate was used to assess survival.

**Results:**

The follow-up rate was 90% (median follow-up: 39.5 months). There were differences in the indication (endocarditis: OA 5 (4.2%) vs non-OA 36 (24%), *p* < 0.0001; dissection: OA 13 (10.9%) vs non-OA 2 (1.3%); *p* = 0.0007). OA patients had less perioperative stroke (1 (1%) vs 15 (10%), *p* = 0.001) and shorter hospital stay (9 vs 12 days, *p* = 0.0004). There were no differences in the mortality (in-hospital: OA 8 (7%) vs non-OA 8 (5%); *p* = 0.6; death at follow-up: OA 5 (5%) vs non-OA 15 (11%); *p* = 0.1). Overall valve performance showed a well-functioning valve in 97.3% at follow-up.

**Conclusion:**

The valve performance showed excellent results regardless of the initial indication. The incidence of stroke was lower in patients receiving an open arch anastomosis using unilateral antegrade cerebral perfusion without elevated mortality or prolonged hospital stay.

## Introduction

The Freestyle® prosthesis (Medtronic plc, Dublin, Ireland) is a porcine aortic root implanted since the 1990s for various pathologies and is often combined with replacement of the ascending aorta in cases of aortopathies [1–4].

Whilst the decision for an open distal anastomosis in elective aortopathies is determined by the size of the aorta and the underlying pathology, the grade of aortic resection in case of dissection can be debateable. The surgical strategy for patients with the dissection extending into the aortic arch but without an intimal tear within the arch itself remains controversial and considering a lower intraoperative risk in the life-threatening situation, a resection of the total aortic arch might be avoided [5]. Hence, hemiarch replacement with resection of the entire lesser curvature and most of the dissected aortic arch wall became a preferred strategy in some institutions for primary repair [6].

Our standard technique for both, elective aortopathies and emergency indications, includes the performance of an open arch anastomosis favoring a hemiarch replacement to avoid complex reinterventions in future, especially if in doubt regarding the quality of the abnormal aorta. As a possible drawback beside the complexity of the combined procedure using the Freestyle® prosthesis, the potentially prolonged cardiopulmonary bypass (CPB) time and the need for hypothermia for selective cerebral perfusion causing a higher risk of bleeding and adverse events are discussed in literature [7–10].

The aim was to assess mid-term outcome regarding prosthetic performance, stroke, reoperations, and survival in various pathologies comparing patients with and without open arch anastomosis focusing on the standardised technique of unilateral antegrade cerebral perfusion through the axillary artery under moderate hypothermia.

## Patients and methods

### Study population

The study protocol was approved by the Local Ethics Committee for the handling and analysis of retrospective data (ID number: 2018–01227, July 20th, 2018). No informed consent was required because the study was untertaken using information consecutively collected in the course of routine care.

Between September 2007 and March 2017, 278 aortic root replacements with the Freestyle® prosthesis were performed by four surgeons at a single centre. Patients were divided in three categories due to the pathology of the aortic root (re-operation vs endocarditis vs dissection). Two groups were identified based on the additional open arch anastomosis resulting in 119 patients (OA) and 159 patients without additional open arch anastomosis (non-OA). Patients with additional valve and coronary artery procedures were also included. Out of the non-OA group, 7 patients were cannulated through the femoral artery and excluded for further analysis. Preoperative baseline characteristics (Table 1), intraoperative and postoperative parameters were extracted from the Dendrite database (Dendrite Clinical Systems Ltd., Reading, UK) and were analysed retrospectively. The follow-up data, including the last echocardiogram, major events, reoperations and death was collected from the hospital software Medfolio® (Nexus AG, Donaueschingen, Germany).

**Table 1:** Patient characteristics

	*n* = 152	*n* = 119	
Patient characteristics	No open anastomosis 56.1%	Open anastomosis 43.9%	p
Gender (female) (%)	35 (23)	25 (21)	0.77
Age (years) at operation (IQR)	64 (56–73)	60 (53–69)	0.02
BMI (IQR)	25.9 (23.8–28.7)	26.2 (24.1–29.0)	0.50
Arterial hypertension (%)	96 (63)	77 (65)	0.80
Nicotin (%)	69 (45)	60 (50)	0.50
COPD/Asthma (%)	11 (7)	6 (5)	0.62
Peripheral arterial occlusive disease (%)	12 (8)	6 (5)	0.46
Dyslipidemia (%)	69 (45)	40 (34)	0.06
Stroke (without/with residuum) (%)	8 (5) / 8 (5)	5 (4) /4 (3)	0.73
Previous myocardial infarction (%)	7 (5)	0 (0)	0.02
Previous cardiac operation (%)	36 (24)	8 (7)	0.0002
**Urgency**			
Elective (%)	121 (80)	96 (81)	0.06
Urgent (%)	22 (14)	9 (8)	
Emergency (%)	9 (6)	14 (11)	
**Ejection fraction (%)**			
- 1 (≥50%)	120 (79)	92 (77)	0.69
- 2 (49–30%)	26 (17)	24 (20)	
- 3 (≤29%)	6 (4)	3 (3)	

### Surgical technique: freestyle® implantation and open arch anastomosis

Patients were cannulated through the right axillary artery either using an 8 mm Dacron® graft, that was sewn beforehand on the artery, or by direct cannulation with the OptiSite® cannula (16–18-20 mm, Edwards Lifesciences Corporation, Irvine, USA) depending on the preference of the surgeon and the quality of the artery. The Freestyle® prosthesis was used as a full root replacement after resection of the native root. The coronary buttons were mobilised and prepared for reimplantation. The left origin of the porcine coronary artery was excised, and the prosthesis orientated towards the native left coronary button to provide a correctly aligned anastomosis. The root prosthesis was implanted using one single 3–0 Toplene® suture line (Santec Medicalprodukte GmbH, Grosshostheim, Germany). After reimplantation of the left coronary button with Prolene® 5–0 (Ethicon, Sommerville, New Jersey, USA), a new ostium or the original right ostium of the porcine prosthesis was prepared for the anastomosis of the right coronary button.

The indication for replacement of the ascending aorta was made in accordance with the current European guidelines for valvular diseases deciding for a replacement in case of an enlarged aortic diameter of 4.5 cm with a combined valve pathology requireing surgical treatment [11, 12]. The decision to perform hemiarch or open arch anastomosis versus only ascending aortic replacement during the root operation was based on the current available guidelines for the treatment of aortic arch disease focusing on the adjacent aneurysm location in the ascending aorta [13]. If the diameter of the distal ascending aorta measured 4.5 cm affecting the proximal arch, hemiarch replacement was performed.

For the open arch anastomosis, unilateral antegrade cerebral perfusion through right axillary access was initiated after reaching hypothermic conditions of 28 °C and by clamping or snaring the brachiocephalic trunc and the left carotid artery leaving the left subclavian artery unblocked. The perfusate was administered with 10–15% of the full flow reaching pressures between 60 and 120 mmHg and a temperature of 22–28 °C. INVOS® Cerebral Oximetry System (Medtronic plc, Dublin, Ireland) was used for transcutanous monitoring of the brain perfusion. CO_2_ insufflation with 2 l/min was used routinely. The aortic clamp was opened in Trendelenburg position and the proximal aortic arch excised as far as indicated. A straight Dacron® prosthesis (sizes between 26 and 32 mm) was anastomosed with 4–0 Prolene to the aortic arch. The brachiocephalic trunc and the left carotid artery were declamped and deaired and the aortic clamp was placed on the graft to reinitiate the general perfusion through the axillary access. As the last step, the anastomosis between the Freestyle® prosthesis and the graft was performed during the rewarming of the patient.

#### Statistical analysis

After extraction of relevant data from our institutional database, statistical analyses were performed using StatsDirect statistical software, version 3.1 (StatsDirect Ltd., Cambridge, UK). Numerical data were expressed as median and interquartile range (IQR). Nominal and categorical variables were given as absolute numbers and proportions (%). In this study most numerical data were non-normally distributed. Non-parametrical test was used for this reason. The Mann–Whitney U-test was used for the comparison of two groups. When comparing numerical data of more than two groups the Kruskal-Wallis test was performed. When a statistically significant difference was shown, multiple comparisons were done using the Dwass-Steel-Critchlow-Fligner method. Nominal data were compared using the test with the Yates correction. The extended version of the Fisher’s exact test (Fisher–Freeman–Halton) was used for nominal and categorical variables in case of small numbers in some categories. Kaplan-Meier estimate was used to assess mid-term survival.

## Results

### Patient characteristics

**Fig. 1:**
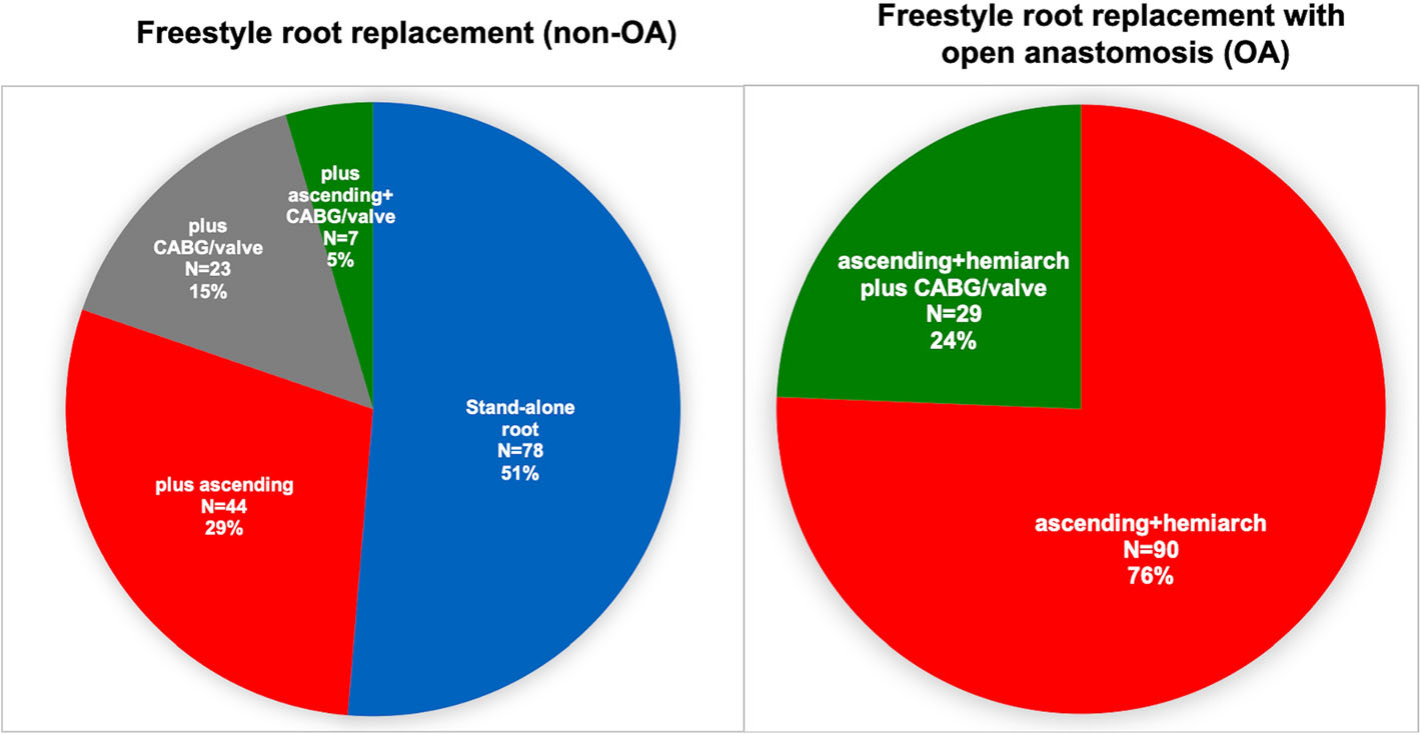
Distribution of additional procedures in total numbers

OA patients had significantly less previous myocardial infarction (*p* = 0.02), previous cardiac operations (*p* = 0.0002) and were younger (60 (53–69) vs 64 (56–73); *p* = 0.02) (Table 1). The distribution for operation urgency was comparable in both groups (*p* = 0.06) with a tendency to more urgent procedures in the non-OA group and more emergencies in the OA group. Patients with additional valve and coronary artery procedures were included and are summarised in Fig. 1.

### Intraoperative parameters

**Fig. 2:**
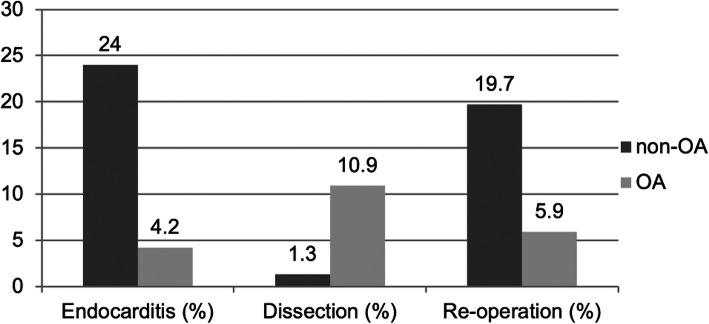
Differences in the indication for operation per group in % (OA vs non-OA) (*p*-values for endocarditis < 0.0001; dissection =0.0007; reoperation =0.001)

The indication for operation was signficantly different with more endocarditis and reoperations in the non-OA group and more dissections in the OA group (Fig. 2). Patients with bicuspid aortic valves received more often a simultaneous open arch anastomosis (OA 60 patients (58%) vs non-OA 45 patients (42%); *p* = 0.0004).

There were significant differences regarding the crossclamp and CPB time in patients with isolated root and ascending replacement compared to patients with additional open arch anastomosis and axillary cannulation (Table 2). For better differentiation, we exluded patients with concomitant procedures and focused on isolated root replacement, root with ascending replacement and root with ascending and open arch anastomosis. Patients with open arch anastomosis and axillary cannulation had shorter CPB and crossclamp times (CPB: OA 116 min (105–135) vs non-OA + ascending 131 min (116–135), *p* = 0.01; crossclamp: OA 88 min (77–102) vs non-OA + ascending 95 min (89–120), *p* = 0.03) compared to patients with root and ascending replacement while the total duration of operation was similar in all groups.

**Table 2:** Cardiopulmonary bypass time for isolated root replacement with or without ascending aorta replacement (+/−) hemiarch in minutes

	Open anastomosis (+ascending aorta) (OA) *n* = 90	Root replacement (+ascending aorta)^a^ (non-OA) *n* = 23	Stand-alone root replacement (non-OA) *n* = 78	p
Cannulation Site	axillary (n = 90)	central (*n* = 14) axillary (n = 9)	central (*n* = 49) axillary (*n* = 29)	
CBP time	116 (105–135)	131 (116–135)	130 (104–152)	0.02^b^
Crossclamp time	88 (77–102)	95 (89–120)	87 (73–112)	0.08^c^
Total duration of OP	250 (219–285)	250 (220–300)	251 (210–335)	0.58

^a^No open anastomosis (+ascending aorta) includes, that the distal aortic anastomosis has been carried out while the aorta was clamped with regular systemic perfusion

^b^OA vs non-OA (+ascending) significant *p* = 0.01 (Kruskal-Wallis: all pairwise comparisons (Conover-Iman))

^c^OA vs non-OA (+ascending) significant *p* = 0.03 (Kruskal-Wallis: all pairwise comparisons (Conover-Iman))

### In-hospital outcome

For the postoperative in-hospital outcome analysis all patients in the non-OA group were included (*n* = 152) and 115 patients in the OA group as 4 patients died intraoperatively due to type A dissections. OA patients had less perioperative stroke (1 patient (0.9%) vs 15 patients (10%), *p* = 0.001). To focus on the cannulation site, three groups were defined for further analysis consisting of 115 OA patients cannulated through axillary artery (1 stroke = 0.9%), 105 non-OA patients cannulated centrally (11 strokes = 10.5%) and 47 non-OA patients cannulated through axillary artery (4 strokes = 8.5%). In the subgroup analysis, there was significantly less stroke in the OA group compared to both non-OA groups (*p* = 0.003). The comparison of both non-OA groups (47 patients cannulated through the axillary artery (4 strokes = 8.5%) and 105 non-OA patients cannulated centrally (11 strokes = 10.5%) showed no significant difference (*p* = 0.71).

OA patients had a tendency to less reoperations on the same admission (*p* = 0.09) (re-sternotomies for bleeding or tamponade (14 patients (12%) vs 35 patients (23%)). There were no differences in perioperative myocardial infarctions (OA 5 (4%) vs non-OA 3 (2%), *p* = 0.3). In-hospital stay was shorter in the OA group (9 vs 12 days, *p* = 0.0004). There were no significant differences in the in-hospital mortality (OA 8 (7%) vs non-OA 8 (5%), *p* = 0.6).

### Follow-up

**Fig. 3:**
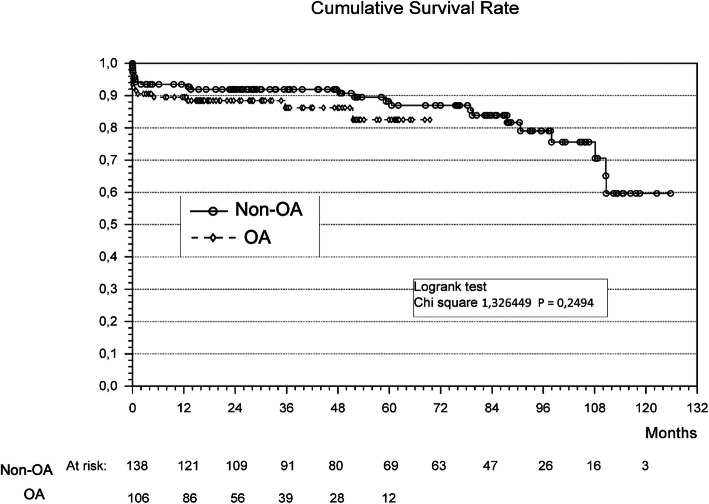
Kaplan Meier Survival Curve showing no differences in the survival rate comparing non-OA and OA patients

The follow-up rate was 90% (loss to follow-up: OA 5 patients, non-OA 6 patients) for a median follow-up of 39.5 months without significant difference in the survival comparing both groups (*p* = 0.24) (Fig. 3). The survival at 1 and 5 years was 89 and 81% in the OA group and 93 and 86% in the non-OA group. One patient per group presented with endocarditis of the root prosthesis (1% vs 1%) in the follow up period (available patient data: 127 non-OA vs 97 OA). There was no significant difference in the death during follow-up (non-OA 15 patients (11%) vs OA 5 patients (5%), *p* = 0.1).

**Fig. 4:**
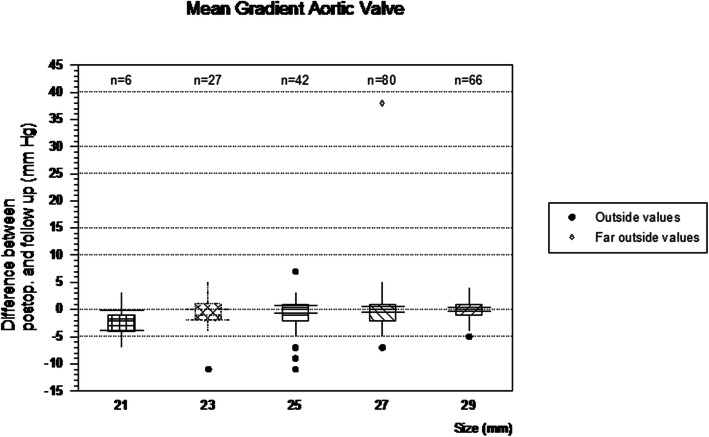
Difference between the postoperative mean gradient compared to the follow-up gradient in mmHg without significant difference in the course from implantation to follow-up comparing the five different implanted sizes of prostheses (*p* = 0.4); *n* = 221

For the calculation of differences in the mean gradients from discharge to follow-up, 250 early postoperative and 221 measurements at follow-up for the whole cohort were available. The postoperative and FU gradients (median + IQR) for the five sizes were as follows: 21 mm: 13 (9–16) vs 13 (7–15) mmHg (*p* = 0.22), 23 mm: 6 (4–8) vs 5 (4–6) mmHg (*p* = 0.09), 25 mm: 5 (4–7) vs 5 (4–7) mmHg (*p* = 0.5), 27 mm: 5 (4–7) vs 5 (3–6) mmHg (*p* = 0.16), 29 mm: 4.5 (3–6) vs 4 (3–5) mmHg (*p* = 0.14); without a significant difference in each of the sizes. Figure 4 illustrates the total number of valves per size of which a FU value was available. The differences between the postoperative and FU mean gradients comparing the five valve sizes were not significant (*p* = 0.4). The valve performance in the whole cohort showed a moderate to severe impairment in 6 of 225 patients (2.7%). The time-to-deterioration calculated in every patient from the implantation date to the follow-up in months was 117 (9.75 years), 105 (8.75 years), 86 (7.2 years), 75 (6.25 years), 51 (4.25 years), 48 (4 years). The last three patients with the shortest time-to-deterioration were 50 years, 64 years and 42 years old at time of operation. In two of the patients, the prosthesis has been implanted into an active endocarditis. The third patient died 4 years after operation due to heart failure having a moderate regurgitation of the valve.

## Discussion

### In-hospital mortality and mid-term survival

In both cohorts, despite different indications for operation, the in-hospital mortality and the survival do not differ significantly, also taking into account that concomitant procedures were carried out more often in the non-OA group. As there are 3 patients at risk after 10 years in the non-OA group, we would like to concentrate on the survival at 1 and 5 years which is compareable with current literature including different pathologies and is not showing a significant difference between both groups [14, 15].

Ennker et al. described a 30-day mortality of 5.4% in isolated root-replacement without concomitant operations on the aorta and 13% mortality of patients with endocarditis as the leading indication [14]. A smaller cohort of 180 patients with active endocarditis was reported with an in-hospital mortality of 11% by Miceli et al. including concomitant procedures, reoperations and interposition grafts for the ascending aorta [4]. Compared to our non-OA group which consists of 24% endocarditis patients, 10.9% of redo-operations and a smaller number of dissections with 1.3% an early mortality of 5% appears quite positive and favourable. Having a closer look at the patients’ cause of death, only three of the eigth patients had an active endocarditis and were operated as emergencies. The other five patients were elective cases, but four also had concomitant bypass operation. In the OA group, four of the eigth patients suffered from dissection, two were reoperations, one had endocarditis and only one patient was electively planned. Focusing on the OA group only, the in-hospital mortality of 7% might reflect the high number of dissections (10.9% = 13 patients). Four of the eigth in-hospital deaths occurred due to acute dissection.

### Operative technique and stroke

We found a lower perioperative stroke rate in the OA group than in the non-OA group (0.9% vs 10%) although the OA group included more patients with dissections. The subanalysis confirmed specifically the finding of a lower stroke rate after axillary cannulation in combination with and without open distal anastomosis. One reason for the lower stroke rate might be the flow dynamics during axillary perfusion leading to the avoidance of manipulation of a sclerotic aorta and a washout of debris [16]. Kaufmann et al. describe a reversed flow in the brachiocephalic trunk caused by central cannulation in their work on positions for outflow cannulas. The negative pressure on the brachiocephalic trunc which is caused by a close position of the aortic cannula causes withdrawal of the blood from the trunc leading to loss in cerebral perfusion [17, 18]. Evidence from various studies supports the beneficial effect of axillary cannulation by preserving antegrade flow in the aortic arch and descending aorta, thereby not only reducing the risk for embolisation, but also facilitating selective cerebral perfusion during circulatory arrest [19].

The technique of axillary cannulation for unilateral antegrade perfusion and open distal anastomosis has been established consistently in our daily practice since 2010, firstly, to eliminate unnecessary general deep hypothermia in type A dissections, and secondly to apply the approach for further pathologies of the ascending aorta and proximal arch by keeping the advantage of extended tissue resection in indicated cases while obtaining a safe cerebral protection [20, 21]. Axillary cannulation has gained increasing popularity, especially in high-volume aortic centres, and has been proven to be safe in type A dissection with involvement of the innominate artery [22]. The European guidelines recommend preferential use of this technique with an evidence class of ‘IIa, level C’ [13].

The overall stroke rate of about 300 patients following the abovementioned set-up for the performance of open distal anastomosis was 7.3% in a cohort of type A dissections [23]. Similar results of a 6% stroke rate were shown by Zierer et al. in a cohort of over 450 patients with dissections, of which 75% received a hemiarch with an open distal anastomosis and uni- or bilateral cerebral perfusion [21]. A study by Matt et al. emphasised the advantage of deep hypothermia (26 °C) and selective bilateral cerebral perfusion with a stroke rate of 4% and early mortality of 2% in a cohort of 178 elective patients receiving hemiarch replacement with open distal anastomosis [24]. The stroke rate of 0.9% in the OA cohort including 10.9% dissections and further procedures is remarkably low, although the data analysis system included transient ischemic attack and cerebral stroke with and without residuum. The low stroke rate in our cohort might be explained by the high number of elective patients who received a root replacement with hemiarch. The standardised use of axillary cannulation in combination with an open distal anastomosis under moderate hypothermia of 28 °C confirms not only to be the approach of choice for dissections, but also in elective cases [25].

The intraoperative procedure time supports the set-up showing similar operating time, but shorter CPB and crossclamp time for patients with axillary cannulation and open anastomosis. While in a metaanalysis of three studies prolonged CPB time during axillary perfusion has been found compared to cannulation of the innominate artery, other sources report no specific disadvantages in CPB time and axillary cannulation [19, 26]. Our experience underlines that despite being techniqually more demanding axillary cannulation and additional hemiarch resection do not lead inevitably to longer operation and CPB times.

### Valve degeneration and gradients

Postoperative gradients measured during follow-up confirmed the low gradient of the Freestyle® prosthesis even in the smaller valve sizes. We detected a low degeneration rate causing moderate to severe impairment of the valve function in 6 patients (2.7%) confirming well-functioning valves in over 97%. Additionally, the mean gradient during the follow-up period remained stable in all valve sizes. The favorable hemodynamic properties with a low thrombogenicity, acceptable durability and reduced transvalvular gradients demonstrate an advantage of the Freestyle® prosthesis [27]. The degeneration rate in literature reviewing long-term outcomes including a follow-up over more than 10 years shows a rate of 6.2 to 7.9% and a linearised detoriation rate 0.2% /100 patient years [14, 15].

### Complications

Both groups showed in the perioperative setting indications for reoperations, while the non-OA group had a tendency to more re-sternotomies. The higher amount of complex and endocarditis patients explains the higher risk of bleeding after deranged coagulation in the non-OA group. Sixteen patients had active endocarditis of which 8 were also reoperations. Further 15 operations were combined procedures of which 13 included coronary artery bypass grafting, therefore receiving early administration of heparin (2 h after operation) and aspirin loading dose (300 mg 3 h after operation). Only 3 patients received a stand-alone root implantation on elective basis and needed re-exploration for bleeding. The re-sternotomies in the OA group included 2 endocarditis patients, 2 dissections, 5 combined procedures and 5 elective root replacements.

Only 1 patient per group had to be reoperated during the late follow-up period because of a prosthetic endocarditis. We could not detect any problems deriving from the distal anastomoses that could have been a reason for reoperation in the non-OA group such as suture line aneurysms or dilatation of the residual aortic tissue, which is not surprising considering the reasonable follow-up period.

#### Limitations

This study has limitations inherent to retrospective study design. Although our follow-up data are 90% complete regarding mortality, data pertaining to other postoperative outcomes such as the valve gradients on the date of follow-up cannot be fully provided as the postoperative echocardiography results were not reported in all patients. The echocardiograms were performed by different investigators in several outpatient clinics.

The study is limited by recording a median follow-up of 39.5 months, although longer term outcomes at 10 and 15 years are relevant to the discussion of reoperations for anastomosis aneurysm, prosthesis failure or the fate of the remaining aorta after ascending or hemiarch replacement. In this context, the follow up period might be too short to detect the development of suture-line aneurysms in patients who received a hemiarch replacement on clamp. The median follow-up of 39.5 months with a maximum follow-up of 125.8 months can be explained by the ranking of the single deaths in the data set which is not normally distributed. Half of the deaths during FU (*n* = 37) appear at up to 1.97 months. Also, the increasing performance of the open anastomosis technique picked up specifically from 2012, when European guidelines were adapted carefully into our clinical practice which correspondingly shows a shorter median FU for the OA group of 26.3 months compared to the non-OA group of 59.5 months.

## Conclusion

The valve performance showed excellent results regardless of the initial indication. The incidence of stroke was lower in the selected group of patients using unilateral antegrade cerebral perfusion under moderate hypothermia with open anastomosis without elevated mortality or prolonged hospital stay.

## Data Availability

The datasets used and/or analysed during the current study are available from the corresponding author on reasonable request.

## Abbreviations

CABG: Coronary artery bypass grafting
CPB: Cardiopulmonary bypass
FU: Follow-up
IQR: Interquartile range
OA: Open anastomosis
OP: Operation
Non-OA: Non open anastomosis
